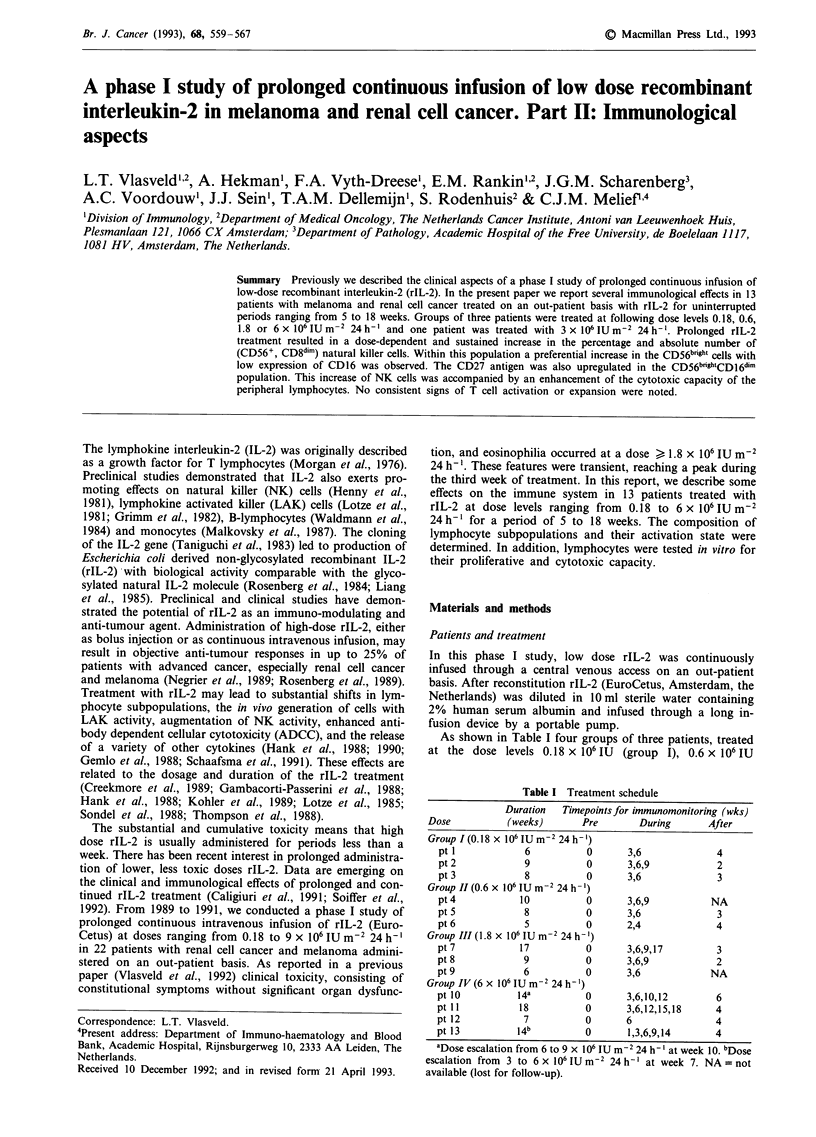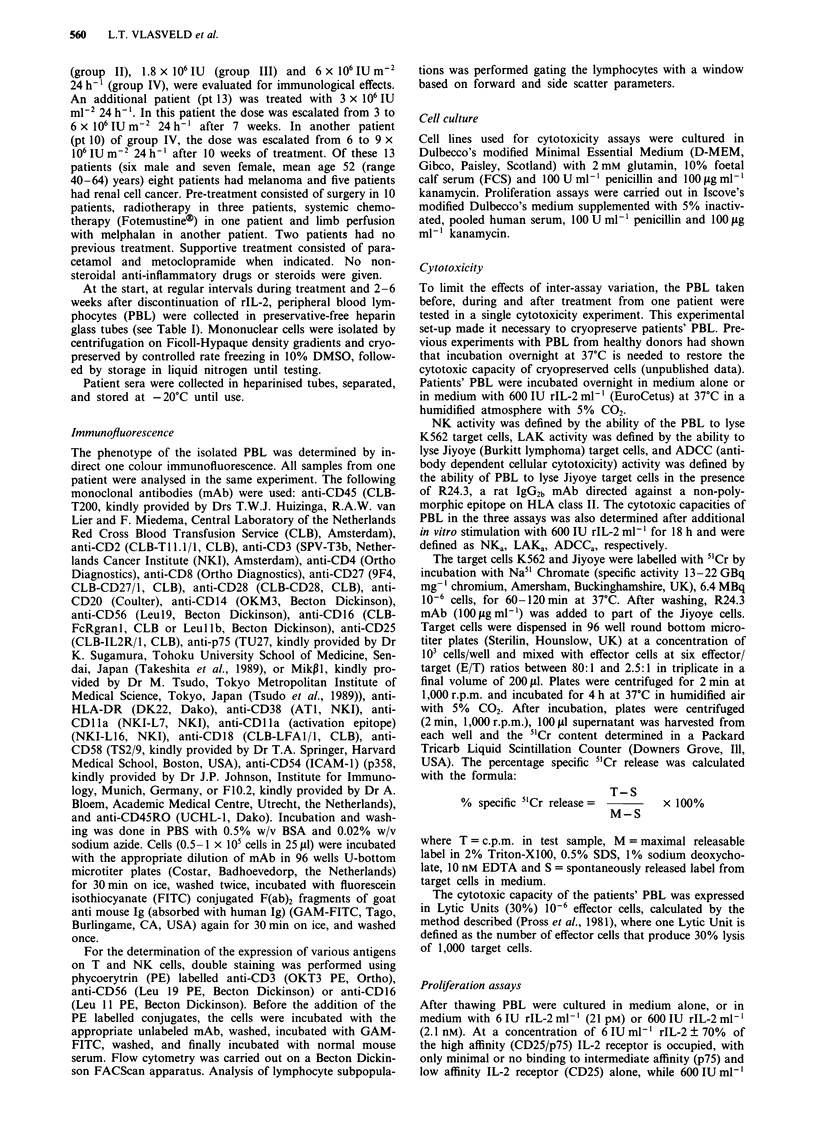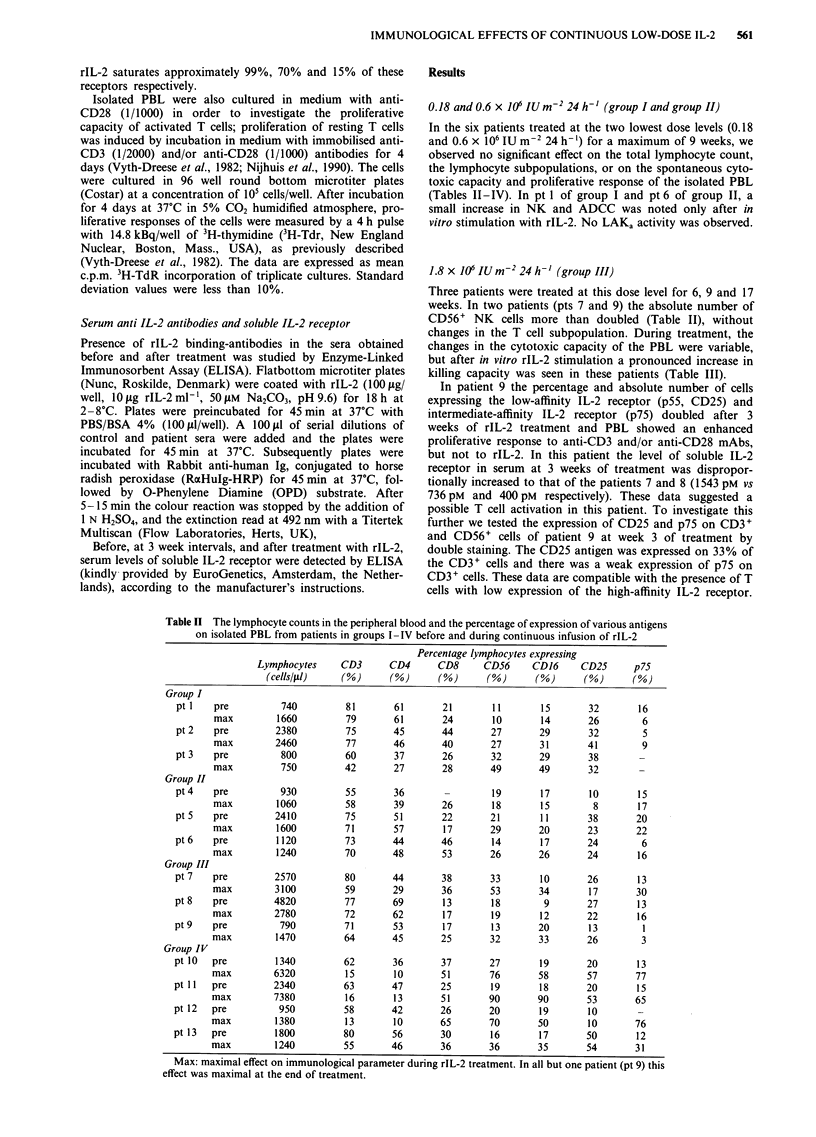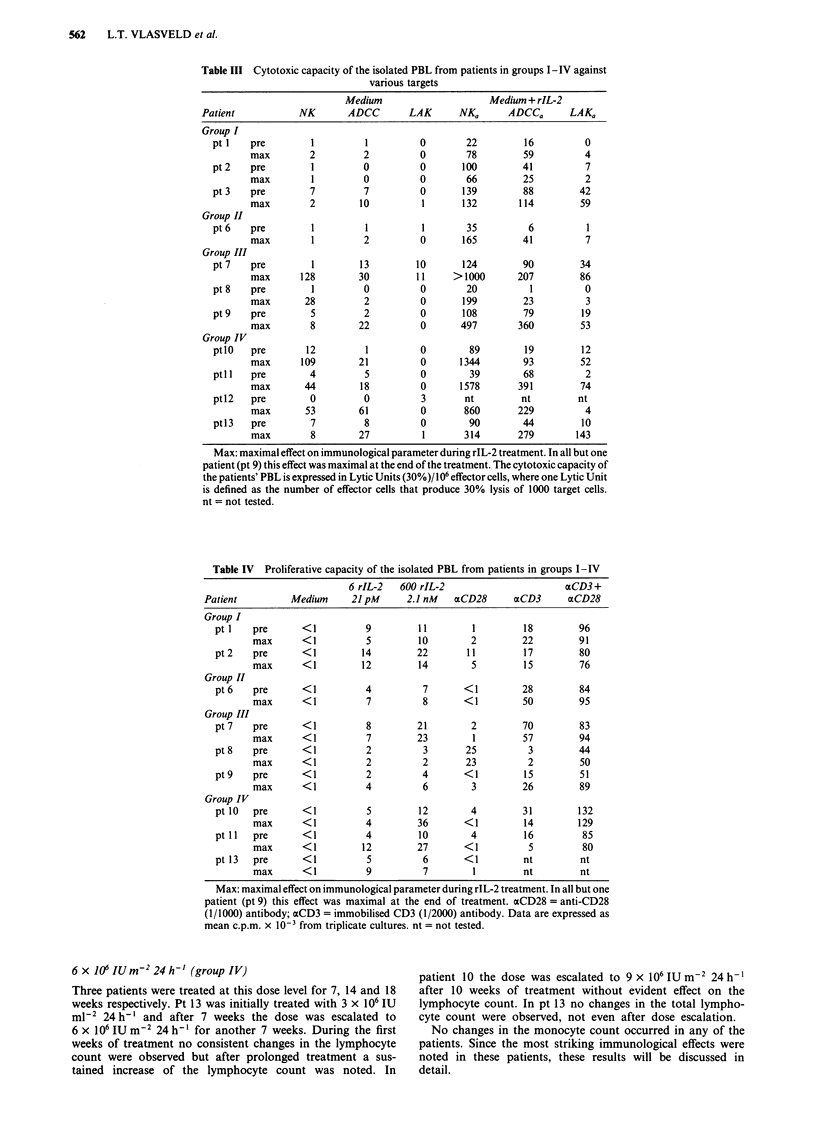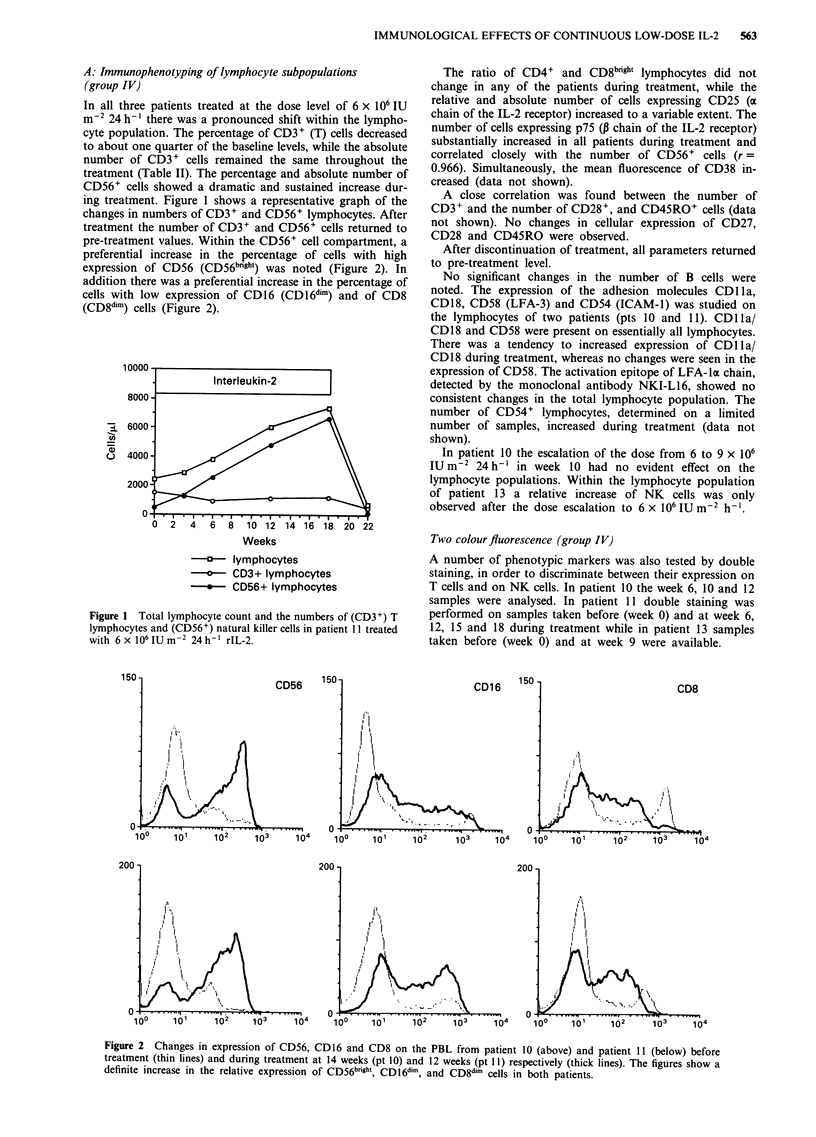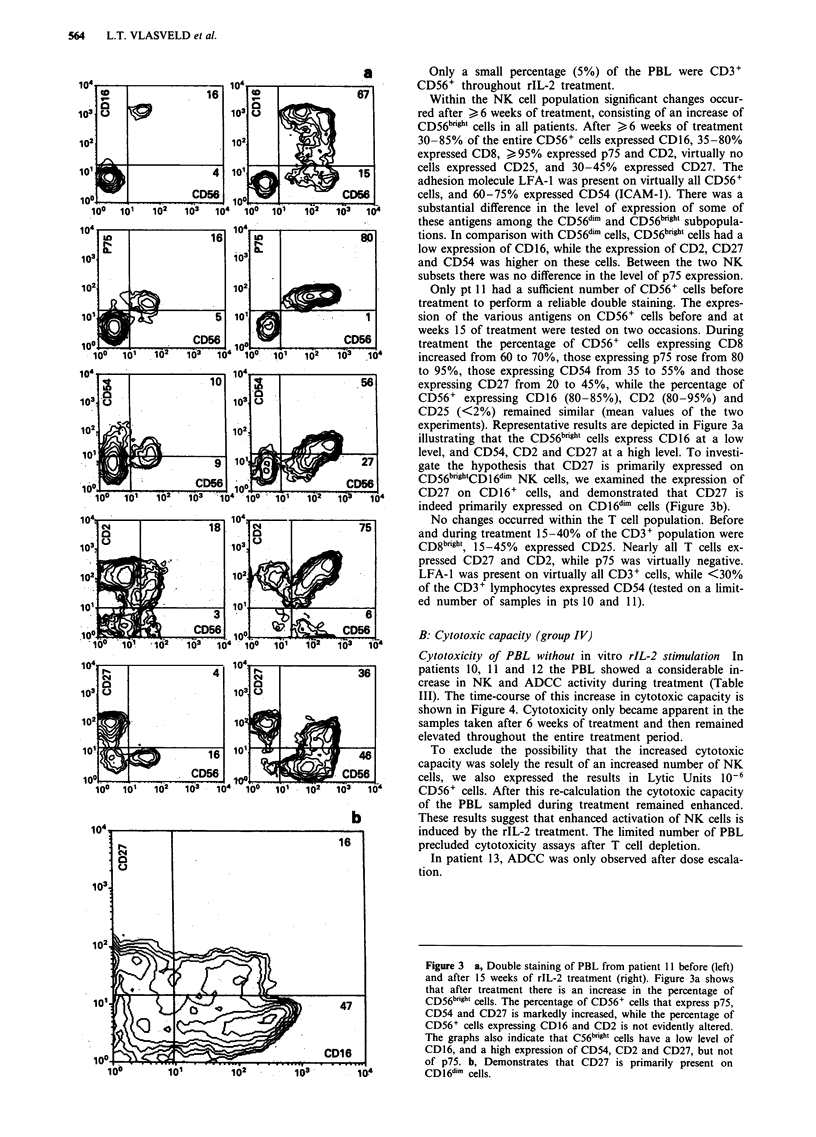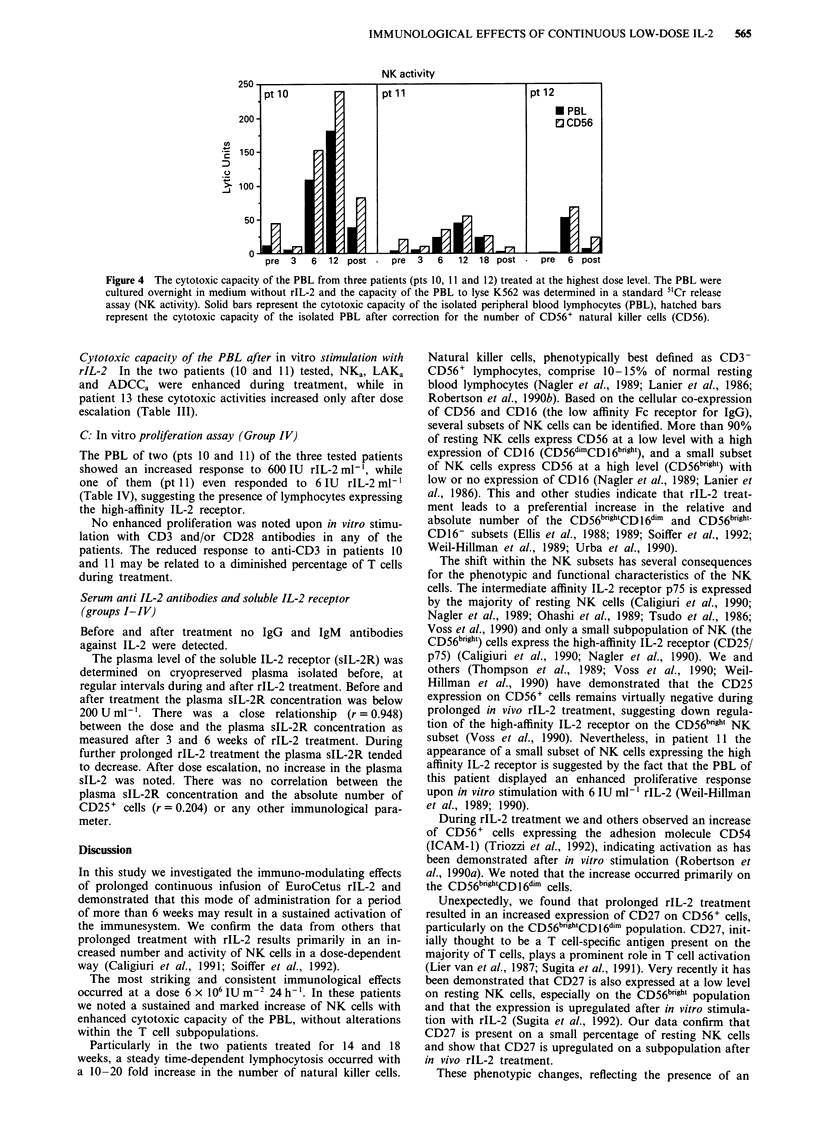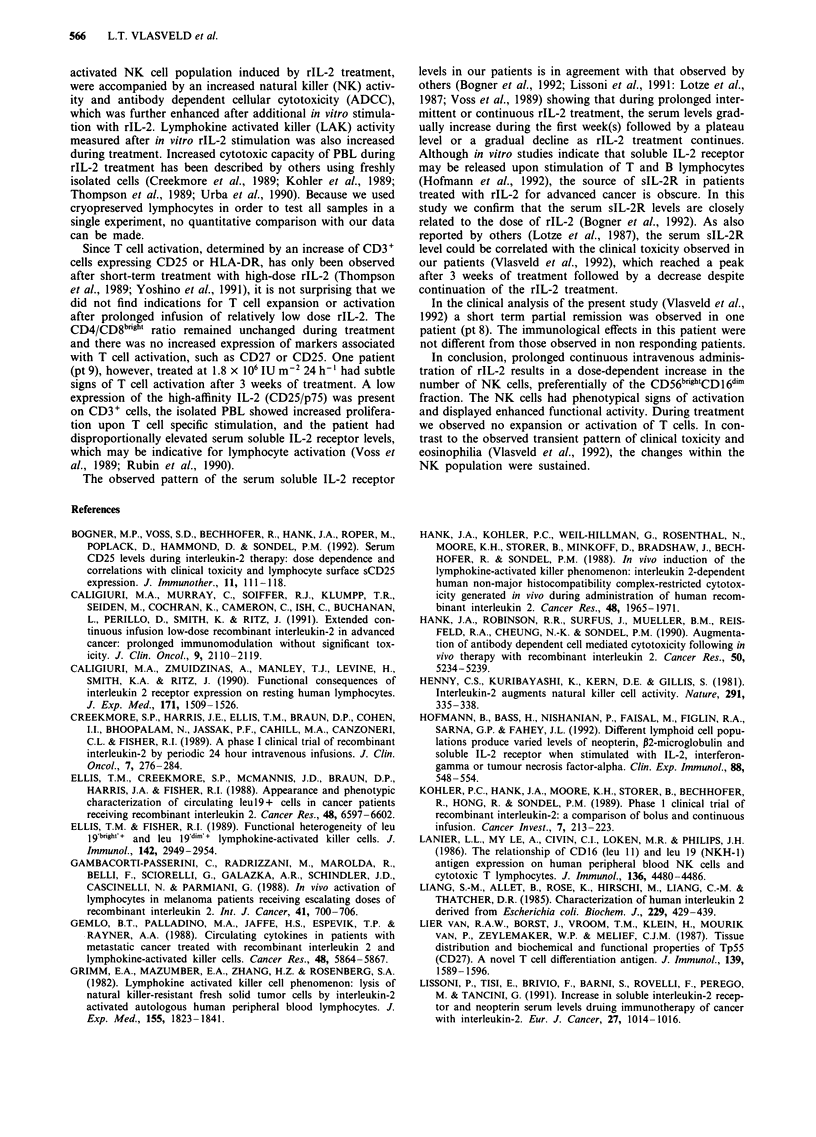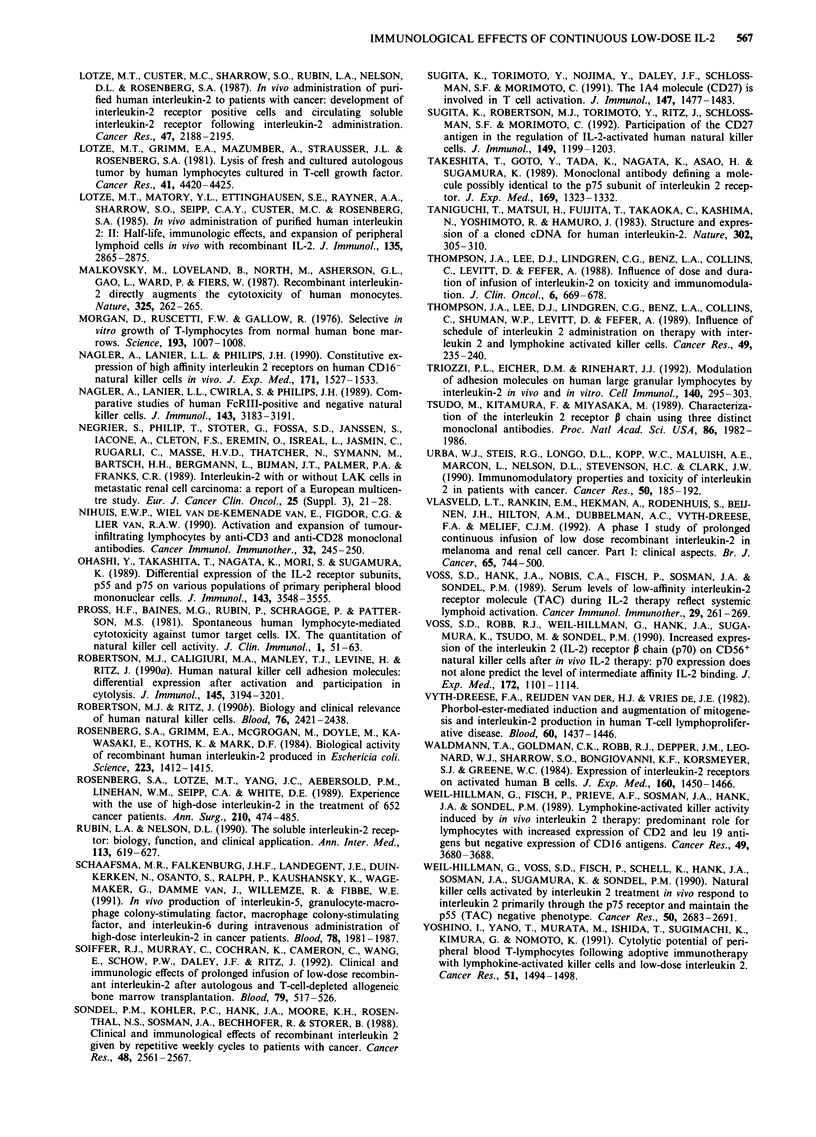# A phase I study of prolonged continuous infusion of low dose recombinant interleukin-2 in melanoma and renal cell cancer. Part II: Immunological aspects.

**DOI:** 10.1038/bjc.1993.386

**Published:** 1993-09

**Authors:** L. T. Vlasveld, A. Hekman, F. A. Vyth-Dreese, E. M. Rankin, J. G. Scharenberg, A. C. Voordouw, J. J. Sein, T. A. Dellemijn, S. Rodenhuis, C. J. Melief

**Affiliations:** Division of Immunology, The Netherlands Cancer Institute, Amsterdam.

## Abstract

Previously we described the clinical aspects of a phase I study of prolonged continuous infusion of low-dose recombinant interleukin-2 (rIL-2). In the present paper we report several immunological effects in 13 patients with melanoma and renal cell cancer treated on an out-patient basis with rIL-2 for uninterrupted periods ranging from 5 to 18 weeks. Groups of three patients were treated at following dose levels 0.18, 0.6, 1.8 or 6 x 10(6) IU m-2 24 h-1 and one patient was treated with 3 x 10(6) IU m-2 24 h-1. Prolonged rIL-2 treatment resulted in a dose-dependent and sustained increase in the percentage and absolute number of (CD56+, CD8dim) natural killer cells. Within this population a preferential increase in the CD56bright cells with low expression of CD16 was observed. The CD27 antigen was also upregulated in the CD56bright CD16dim population. This increase of NK cells was accompanied by an enhancement of the cytotoxic capacity of the peripheral lymphocytes. No consistent signs of T cell activation or expansion were noted.


Br. J. Cancer (1993), 68, 559-567 © Macmillan Press Ltd., 1993

### A phase I study of prolonged continuous infusion of low dose recombinant interleukin-2 in melanoma and renal cell cancer. Part II: Immunological aspects

L.T. Vlasveld[1,2], A. Hekman[1], F.A. Vyth-Dreese[1], E.M. Rankin[1,2], J.G.M. Scharenberg[3], A.C. Voordouw[1], J.J. Sein[1], T.A.M. Dellemijn[1], S. Rodenhuis[2] & C.J.M. Melief[1,4]

[1]*Division of Immunology,* [2]*Department of Medical Oncology, The Netherlands Cancer Institute, Antoni van Leeuwenhoek Huis, Plesmanlaan 121, 1066 CX Amsterdam;* [3]*Department of Pathology, Academic Hospital of the Free University, de Boelelaan 1117, 1081 HV, Amsterdam, The Netherlands.*

**Summary** Previously we described the clinical aspects of a phase I study of prolonged continuous infusion of low-dose recombinant interleukin-2 (rIL-2). In the present paper we report several immunological effects in 13 patients with melanoma and renal cell cancer treated on an out-patient basis with rIL-2 for uninterrupted periods ranging from 5 to 18 weeks. Groups of three patients were treated at following dose levels 0.18, 0.6, 1.8 or $6 \times 10^6$ IU m$^{-2}$ 24 h$^{-1}$ and one patient was treated with $3 \times 10^6$ IU m$^{-2}$ 24 h$^{-1}$. Prolonged rIL-2 treatment resulted in a dose-dependent and sustained increase in the percentage and absolute number of ($CD56^+$, $CD8^{dim}$) natural killer cells. Within this population a preferential increase in the $CD56^{bright}$ cells with low expression of CD16 was observed. The CD27 antigen was also upregulated in the $CD56^{bright}CD16^{dim}$ population. This increase of NK cells was accompanied by an enhancement of the cytotoxic capacity of the peripheral lymphocytes. No consistent signs of T cell activation or expansion were noted.

The lymphokine interleukin-2 (IL-2) was originally described as a growth factor for T lymphocytes (Morgan *et al.*, 1976). Preclinical studies demonstrated that IL-2 also exerts promoting effects on natural killer (NK) cells (Henny *et al.*, 1981), lymphokine activated killer (LAK) cells (Lotze *et al.*, 1981; Grimm *et al.*, 1982), B-lymphocytes (Waldmann *et al.*, 1984) and monocytes (Malkovsky *et al.*, 1987). The cloning of the IL-2 gene (Taniguchi *et al.*, 1983) led to production of *Escherichia coli* derived non-glycosylated recombinant IL-2 (rIL-2) with biological activity comparable with the glycosylated natural IL-2 molecule (Rosenberg *et al.*, 1984; Liang *et al.*, 1985). Preclinical and clinical studies have demonstrated the potential of rIL-2 as an immuno-modulating and anti-tumour agent. Administration of high-dose rIL-2, either as bolus injection or as continuous intravenous infusion, may result in objective anti-tumour responses in up to 25% of patients with advanced cancer, especially renal cell cancer and melanoma (Negrier *et al.*, 1989; Rosenberg *et al.*, 1989). Treatment with rIL-2 may lead to substantial shifts in lymphocyte subpopulations, the *in vivo* generation of cells with LAK activity, augmentation of NK activity, enhanced antibody dependent cellular cytotoxicity (ADCC), and the release of a variety of other cytokines (Hank *et al.*, 1988; 1990; Gemlo *et al.*, 1988; Schaafsma *et al.*, 1991). These effects are related to the dosage and duration of the rIL-2 treatment (Creekmore *et al.*, 1989; Gambacorti-Passerini *et al.*, 1988; Hank *et al.*, 1988; Kohler *et al.*, 1989; Lotze *et al.*, 1985; Sondel *et al.*, 1988; Thompson *et al.*, 1988).

The substantial and cumulative toxicity means that high dose rIL-2 is usually administered for periods less than a week. There has been recent interest in prolonged administration of lower, less toxic doses rIL-2. Data are emerging on the clinical and immunological effects of prolonged and continued rIL-2 treatment (Caligiuri *et al.*, 1991; Soiffer *et al.*, 1992). From 1989 to 1991, we conducted a phase I study of prolonged continuous intravenous infusion of rIL-2 (EuroCetus) at doses ranging from 0.18 to $9 \times 10^6$ IU m$^{-2}$ 24 h$^{-1}$ in 22 patients with renal cell cancer and melanoma administered on an out-patient basis. As reported in a previous paper (Vlasveld *et al.*, 1992) clinical toxicity, consisting of constitutional symptoms without significant organ dysfunction, and eosinophilia occurred at a dose $\geqslant 1.8 \times 10^6$ IU m$^{-2}$ 24 h$^{-1}$. These features were transient, reaching a peak during the third week of treatment. In this report, we describe some effects on the immune system in 13 patients treated with rIL-2 at dose levels ranging from 0.18 to $6 \times 10^6$ IU m$^{-2}$ 24 h$^{-1}$ for a period of 5 to 18 weeks. The composition of lymphocyte subpopulations and their activation state were determined. In addition, lymphocytes were tested *in vitro* for their proliferative and cytotoxic capacity.

Correspondence: L.T. Vlasveld.
[4]Present address: Department of Immuno-haematology and Blood Bank, Academic Hospital, Rijnsburgerweg 10, 2333 AA Leiden, The Netherlands.
Received 10 December 1992; and in revised form 21 April 1993.

### Materials and methods

### *Patients and treatment*

In this phase I study, low dose rIL-2 was continuously infused through a central venous access on an out-patient basis. After reconstitution rIL-2 (EuroCetus, Amsterdam, the Netherlands) was diluted in 10 ml sterile water containing 2% human serum albumin and infused through a long infusion device by a portable pump.

As shown in Table I four groups of three patients, treated at the dose levels $0.18 \times 10^6$ IU (group I), $0.6 \times 10^6$ IU

**Table I** Treatment schedule

| Dose | Duration (weeks) | Timepoints for immunomonitoring (wks) Pre | During | After |
|---|---|---|---|---|
| *Group I* ($0.18 \times 10^6$ IU m$^{-2}$ 24 h$^{-1}$) | | | | |
| pt 1 | 6 | 0 | 3,6 | 4 |
| pt 2 | 9 | 0 | 3,6,9 | 2 |
| pt 3 | 8 | 0 | 3,6 | 3 |
| *Group II* ($0.6 \times 10^6$ IU m$^{-2}$ 24 h$^{-1}$) | | | | |
| pt 4 | 10 | 0 | 3,6,9 | NA |
| pt 5 | 8 | 0 | 3,6 | 3 |
| pt 6 | 5 | 0 | 2,4 | 4 |
| *Group III* ($1.8 \times 10^6$ IU m$^{-2}$ 24 h$^{-1}$) | | | | |
| pt 7 | 17 | 0 | 3,6,9,17 | 3 |
| pt 8 | 9 | 0 | 3,6,9 | 2 |
| pt 9 | 6 | 0 | 3,6 | NA |
| *Group IV* ($6 \times 10^6$ IU m$^{-2}$ 24 h$^{-1}$) | | | | |
| pt 10 | 14[a] | 0 | 3,6,10,12 | 6 |
| pt 11 | 18 | 0 | 3,6,12,15,18 | 4 |
| pt 12 | 7 | 0 | 6 | 4 |
| pt 13 | 14[b] | 0 | 1,3,6,9,14 | 4 |

[a]Dose escalation from 6 to $9 \times 10^6$ IU m$^{-2}$ 24 h$^{-1}$ at week 10. [b]Dose escalation from 3 to $6 \times 10^6$ IU m$^{-2}$ 24 h$^{-1}$ at week 7. NA = not available (lost for follow-up).

(group II), $1.8 \times 10^6$ IU (group III) and $6 \times 10^6$ IU $m^{-2}$ 24 $h^{-1}$ (group IV), were evaluated for immunological effects. An additional patient (pt 13) was treated with $3 \times 10^6$ IU $ml^{-2}$ 24 $h^{-1}$. In this patient the dose was escalated from 3 to $6 \times 10^6$ IU $m^{-2}$ 24 $h^{-1}$ after 7 weeks. In another patient (pt 10) of group IV, the dose was escalated from 6 to $9 \times 10^6$ IU $m^{-2}$ 24 $h^{-1}$ after 10 weeks of treatment. Of these 13 patients (six male and seven female, mean age 52 (range 40–64) years) eight patients had melanoma and five patients had renal cell cancer. Pre-treatment consisted of surgery in 10 patients, radiotherapy in three patients, systemic chemotherapy (Fotemustine®) in one patient and limb perfusion with melphalan in another patient. Two patients had no previous treatment. Supportive treatment consisted of paracetamol and metoclopramide when indicated. No non-steroidal anti-inflammatory drugs or steroids were given.

At the start, at regular intervals during treatment and 2–6 weeks after discontinuation of rIL-2, peripheral blood lymphocytes (PBL) were collected in preservative-free heparin glass tubes (see Table I). Mononuclear cells were isolated by centrifugation on Ficoll-Hypaque density gradients and cryopreserved by controlled rate freezing in 10% DMSO, followed by storage in liquid nitrogen until testing.

Patient sera were collected in heparinised tubes, separated, and stored at −20°C until use.

### *Immunofluorescence*

The phenotype of the isolated PBL was determined by indirect one colour immunofluorescence. All samples from one patient were analysed in the same experiment. The following monoclonal antibodies (mAb) were used: anti-CD45 (CLB-T200, kindly provided by Drs T.W.J. Huizinga, R.A.W. van Lier and F. Miedema, Central Laboratory of the Netherlands Red Cross Blood Transfusion Service (CLB), Amsterdam), anti-CD2 (CLB-T11.1/1, CLB), anti-CD3 (SPV-T3b, Netherlands Cancer Institute (NKI), Amsterdam), anti-CD4 (Ortho Diagnostics), anti-CD8 (Ortho Diagnostics), anti-CD27 (9F4, CLB-CD27/1, CLB), anti-CD28 (CLB-CD28, CLB), anti-CD20 (Coulter), anti-CD14 (OKM3, Becton Dickinson), anti-CD56 (Leu19, Becton Dickinson), anti-CD16 (CLB-FcRgran1, CLB or Leu11b, Becton Dickinson), anti-CD25 (CLB-IL2R/1, CLB), anti-p75 (TU27, kindly provided by Dr K. Sugamura, Tohoku University School of Medicine, Sendai, Japan (Takeshita *et al.*, 1989), or Mikβ1, kindly provided by Dr M. Tsudo, Tokyo Metropolitan Institute of Medical Science, Tokyo, Japan (Tsudo *et al.*, 1989)), anti-HLA-DR (DK22, Dako), anti-CD38 (AT1, NKI), anti-CD11a (NKI-L7, NKI), anti-CD11a (activation epitope) (NKI-L16, NKI), anti-CD18 (CLB-LFA1/1, CLB), anti-CD58 (TS2/9, kindly provided by Dr T.A. Springer, Harvard Medical School, Boston, USA), anti-CD54 (ICAM-1) (p358, kindly provided by Dr J.P. Johnson, Institute for Immunology, Munich, Germany, or F10.2, kindly provided by Dr A. Bloem, Academic Medical Centre, Utrecht, the Netherlands), and anti-CD45RO (UCHL-1, Dako). Incubation and washing was done in PBS with 0.5% w/v BSA and 0.02% w/v sodium azide. Cells ($0.5-1 \times 10^5$ cells in 25 µl) were incubated with the appropriate dilution of mAb in 96 wells U-bottom microtiter plates (Costar, Badhoevedorp, the Netherlands) for 30 min on ice, washed twice, incubated with fluorescein isothiocyanate (FITC) conjugated $F(ab)_2$ fragments of goat anti mouse Ig (absorbed with human Ig) (GAM-FITC, Tago, Burlingame, CA, USA) again for 30 min on ice, and washed once.

For the determination of the expression of various antigens on T and NK cells, double staining was performed using phycoerytrin (PE) labelled anti-CD3 (OKT3 PE, Ortho), anti-CD56 (Leu 19 PE, Becton Dickinson) or anti-CD16 (Leu 11 PE, Becton Dickinson). Before the addition of the PE labelled conjugates, the cells were incubated with the appropriate unlabeled mAb, washed, incubated with GAM-FITC, washed, and finally incubated with normal mouse serum. Flow cytometry was carried out on a Becton Dickinson FACScan apparatus. Analysis of lymphocyte subpopulations was performed gating the lymphocytes with a window based on forward and side scatter parameters.

### *Cell culture*

Cell lines used for cytotoxicity assays were cultured in Dulbecco's modified Minimal Essential Medium (D-MEM, Gibco, Paisley, Scotland) with 2 mM glutamin, 10% foetal calf serum (FCS) and 100 U $ml^{-1}$ penicillin and 100 µg $ml^{-1}$ kanamycin. Proliferation assays were carried out in Iscove's modified Dulbecco's medium supplemented with 5% inactivated, pooled human serum, 100 U $ml^{-1}$ penicillin and 100 µg $ml^{-1}$ kanamycin.

### *Cytotoxicity*

To limit the effects of inter-assay variation, the PBL taken before, during and after treatment from one patient were tested in a single cytotoxicity experiment. This experimental set-up made it necessary to cryopreserve patients' PBL. Previous experiments with PBL from healthy donors had shown that incubation overnight at 37°C is needed to restore the cytotoxic capacity of cryopreserved cells (unpublished data). Patients' PBL were incubated overnight in medium alone or in medium with 600 IU rIL-2 $ml^{-1}$ (EuroCetus) at 37°C in a humidified atmosphere with 5% $CO_2$.

NK activity was defined by the ability of the PBL to lyse K562 target cells, LAK activity was defined by the ability to lyse Jiyoye (Burkitt lymphoma) target cells, and ADCC (antibody dependent cellular cytotoxicity) activity was defined by the ability of PBL to lyse Jiyoye target cells in the presence of R24.3, a rat $IgG_{2b}$ mAb directed against a non-polymorphic epitope on HLA class II. The cytotoxic capacities of PBL in the three assays was also determined after additional *in vitro* stimulation with 600 IU rIL-2 $ml^{-1}$ for 18 h and were defined as $NK_a$, $LAK_a$, $ADCC_a$, respectively.

The target cells K562 and Jiyoye were labelled with $^{51}Cr$ by incubation with $Na^{51}$ Chromate (specific activity 13–22 GBq $mg^{-1}$ chromium, Amersham, Buckinghamshire, UK), 6.4 MBq $10^{-6}$ cells, for 60–120 min at 37°C. After washing, R24.3 mAb (100 µg $ml^{-1}$) was added to part of the Jiyoye cells. Target cells were dispensed in 96 well round bottom microtiter plates (Sterilin, Hounslow, UK) at a concentration of $10^3$ cells/well and mixed with effector cells at six effector/target (E/T) ratios between 80:1 and 2.5:1 in triplicate in a final volume of 200 µl. Plates were centrifuged for 2 min at 1,000 r.p.m. and incubated for 4 h at 37°C in humidified air with 5% $CO_2$. After incubation, plates were centrifuged (2 min, 1,000 r.p.m.), 100 µl supernatant was harvested from each well and the $^{51}Cr$ content determined in a Packard Tricarb Liquid Scintillation Counter (Downers Grove, Ill, USA). The percentage specific $^{51}Cr$ release was calculated with the formula:

$$\% \text{ specific } ^{51}\text{Cr release} = \frac{T-S}{M-S} \times 100\%$$

where T = c.p.m. in test sample, M = maximal releasable label in 2% Triton-X100, 0.5% SDS, 1% sodium deoxycholate, 10 nM EDTA and S = spontaneously released label from target cells in medium.

The cytotoxic capacity of the patients' PBL was expressed in Lytic Units (30%) $10^{-6}$ effector cells, calculated by the method described (Pross *et al.*, 1981), where one Lytic Unit is defined as the number of effector cells that produce 30% lysis of 1,000 target cells.

### *Proliferation assays*

After thawing PBL were cultured in medium alone, or in medium with 6 IU rIL-2 $ml^{-1}$ (21 pM) or 600 IU rIL-2 $ml^{-1}$ (2.1 nM). At a concentration of 6 IU $ml^{-1}$ rIL-2 ± 70% of the high affinity (CD25/p75) IL-2 receptor is occupied, with only minimal or no binding to intermediate affinity (p75) and low affinity IL-2 receptor (CD25) alone, while 600 IU $ml^{-1}$

rIL-2 saturates approximately 99%, 70% and 15% of these receptors respectively.

Isolated PBL were also cultured in medium with anti-CD28 (1/1000) in order to investigate the proliferative capacity of activated T cells; proliferation of resting T cells was induced by incubation in medium with immobilised anti-CD3 (1/2000) and/or anti-CD28 (1/1000) antibodies for 4 days (Vyth-Dreese *et al.*, 1982; Nijhuis *et al.*, 1990). The cells were cultured in 96 well round bottom microtiter plates (Costar) at a concentration of $10^5$ cells/well. After incubation for 4 days at 37°C in 5% $CO_2$ humidified atmosphere, proliferative responses of the cells were measured by a 4 h pulse with 14.8 kBq/well of $^3$H-thymidine ($^3$H-Tdr, New England Nuclear, Boston, Mass., USA), as previously described (Vyth-Dreese *et al.*, 1982). The data are expressed as mean c.p.m. $^3$H-TdR incorporation of triplicate cultures. Standard deviation values were less than 10%.

*Serum anti IL-2 antibodies and soluble IL-2 receptor*

Presence of rIL-2 binding-antibodies in the sera obtained before and after treatment was studied by Enzyme-Linked Immunosorbent Assay (ELISA). Flatbottom microtiter plates (Nunc, Roskilde, Denmark) were coated with rIL-2 (100 µg/well, 10 µg rIL-2 $ml^{-1}$, 50 µM $Na_2CO_3$, pH 9.6) for 18 h at 2–8°C. Plates were preincubated for 45 min at 37°C with PBS/BSA 4% (100 µl/well). A 100 µl of serial dilutions of control and patient sera were added and the plates were incubated for 45 min at 37°C. Subsequently plates were incubated with Rabbit anti-human Ig, conjugated to horse radish peroxidase (RαHuIg-HRP) for 45 min at 37°C, followed by O-Phenylene Diamine (OPD) substrate. After 5–15 min the colour reaction was stopped by the addition of 1 N $H_2SO_4$, and the extinction read at 492 nm with a Titertek Multiscan (Flow Laboratories, Herts, UK),

Before, at 3 week intervals, and after treatment with rIL-2, serum levels of soluble IL-2 receptor were detected by ELISA (kindly provided by EuroGenetics, Amsterdam, the Netherlands), according to the manufacturer's instructions.

### Results

*0.18 and 0.6 × $10^6$ IU $m^{-2}$ 24 $h^{-1}$ (group I and group II)*

In the six patients treated at the two lowest dose levels (0.18 and 0.6 × $10^6$ IU $m^{-2}$ 24 $h^{-1}$) for a maximum of 9 weeks, we observed no significant effect on the total lymphocyte count, the lymphocyte subpopulations, or on the spontaneous cytotoxic capacity and proliferative response of the isolated PBL (Tables II–IV). In pt 1 of group I and pt 6 of group II, a small increase in NK and ADCC was noted only after *in vitro* stimulation with rIL-2. No $LAK_a$ activity was observed.

*1.8 × $10^6$ IU $m^{-2}$ 24 $h^{-1}$ (group III)*

Three patients were treated at this dose level for 6, 9 and 17 weeks. In two patients (pts 7 and 9) the absolute number of $CD56^+$ NK cells more than doubled (Table II), without changes in the T cell subpopulation. During treatment, the changes in the cytotoxic capacity of the PBL were variable, but after *in vitro* rIL-2 stimulation a pronounced increase in killing capacity was seen in these patients (Table III).

In patient 9 the percentage and absolute number of cells expressing the low-affinity IL-2 receptor (p55, CD25) and intermediate-affinity IL-2 receptor (p75) doubled after 3 weeks of rIL-2 treatment and PBL showed an enhanced proliferative response to anti-CD3 and/or anti-CD28 mAbs, but not to rIL-2. In this patient the level of soluble IL-2 receptor in serum at 3 weeks of treatment was disproportionally increased to that of the patients 7 and 8 (1543 pM *vs* 736 pM and 400 pM respectively). These data suggested a possible T cell activation in this patient. To investigate this further we tested the expression of CD25 and p75 on $CD3^+$ and $CD56^+$ cells of patient 9 at week 3 of treatment by double staining. The CD25 antigen was expressed on 33% of the $CD3^+$ cells and there was a weak expression of p75 on $CD3^+$ cells. These data are compatible with the presence of T cells with low expression of the high-affinity IL-2 receptor.

**Table II** The lymphocyte counts in the peripheral blood and the percentage of expression of various antigens on isolated PBL from patients in groups I–IV before and during continuous infusion of rIL-2

| | | Lymphocytes (cells/µl) | Percentage lymphocytes expressing CD3 (%) | CD4 (%) | CD8 (%) | CD56 (%) | CD16 (%) | CD25 (%) | p75 (%) |
|---|---|---|---|---|---|---|---|---|---|
| *Group I* | | | | | | | | | |
| pt 1 | pre | 740 | 81 | 61 | 21 | 11 | 15 | 32 | 16 |
| | max | 1660 | 79 | 61 | 24 | 10 | 14 | 26 | 6 |
| pt 2 | pre | 2380 | 75 | 45 | 44 | 27 | 29 | 32 | 5 |
| | max | 2460 | 77 | 46 | 40 | 27 | 31 | 41 | 9 |
| pt 3 | pre | 800 | 60 | 37 | 26 | 32 | 29 | 38 | – |
| | max | 750 | 42 | 27 | 28 | 49 | 49 | 32 | – |
| *Group II* | | | | | | | | | |
| pt 4 | pre | 930 | 55 | 36 | – | 19 | 17 | 10 | 15 |
| | max | 1060 | 58 | 39 | 26 | 18 | 15 | 8 | 17 |
| pt 5 | pre | 2410 | 75 | 51 | 22 | 21 | 11 | 38 | 20 |
| | max | 1600 | 71 | 57 | 17 | 29 | 20 | 23 | 22 |
| pt 6 | pre | 1120 | 73 | 44 | 46 | 14 | 17 | 24 | 6 |
| | max | 1240 | 70 | 48 | 53 | 26 | 26 | 24 | 16 |
| *Group III* | | | | | | | | | |
| pt 7 | pre | 2570 | 80 | 44 | 38 | 33 | 10 | 26 | 13 |
| | max | 3100 | 59 | 29 | 36 | 53 | 34 | 17 | 30 |
| pt 8 | pre | 4820 | 77 | 69 | 13 | 18 | 9 | 27 | 13 |
| | max | 2780 | 72 | 62 | 17 | 19 | 12 | 22 | 16 |
| pt 9 | pre | 790 | 71 | 53 | 17 | 13 | 20 | 13 | 1 |
| | max | 1470 | 64 | 45 | 25 | 32 | 33 | 26 | 3 |
| *Group IV* | | | | | | | | | |
| pt 10 | pre | 1340 | 62 | 36 | 37 | 27 | 19 | 20 | 13 |
| | max | 6320 | 15 | 10 | 51 | 76 | 58 | 57 | 77 |
| pt 11 | pre | 2340 | 63 | 47 | 25 | 19 | 18 | 20 | 15 |
| | max | 7380 | 16 | 13 | 51 | 90 | 90 | 53 | 65 |
| pt 12 | pre | 950 | 58 | 42 | 26 | 20 | 19 | 10 | – |
| | max | 1380 | 13 | 10 | 65 | 70 | 50 | 10 | 76 |
| pt 13 | pre | 1800 | 80 | 56 | 30 | 16 | 17 | 50 | 12 |
| | max | 1240 | 55 | 46 | 36 | 36 | 35 | 54 | 31 |

Max: maximal effect on immunological parameter during rIL-2 treatment. In all but one patient (pt 9) this effect was maximal at the end of treatment.

**Table III** Cytotoxic capacity of the isolated PBL from patients in groups I–IV against various targets

| *Patient* | | *NK* | *Medium* *ADCC* | *LAK* | *$NK_a$* | *Medium + rIL-2* *$ADCC_a$* | *$LAK_a$* |
|---|---|---|---|---|---|---|---|
| *Group I* | | | | | | | |
| pt 1 | pre | 1 | 1 | 0 | 22 | 16 | 0 |
| | max | 2 | 2 | 0 | 78 | 59 | 4 |
| pt 2 | pre | 1 | 0 | 0 | 100 | 41 | 7 |
| | max | 1 | 0 | 0 | 66 | 25 | 2 |
| pt 3 | pre | 7 | 7 | 0 | 139 | 88 | 42 |
| | max | 2 | 10 | 1 | 132 | 114 | 59 |
| *Group II* | | | | | | | |
| pt 6 | pre | 1 | 1 | 1 | 35 | 6 | 1 |
| | max | 1 | 2 | 0 | 165 | 41 | 7 |
| *Group III* | | | | | | | |
| pt 7 | pre | 1 | 13 | 10 | 124 | 90 | 34 |
| | max | 128 | 30 | 11 | >1000 | 207 | 86 |
| pt 8 | pre | 1 | 0 | 0 | 20 | 1 | 0 |
| | max | 28 | 2 | 0 | 199 | 23 | 3 |
| pt 9 | pre | 5 | 2 | 0 | 108 | 79 | 19 |
| | max | 8 | 22 | 0 | 497 | 360 | 53 |
| *Group IV* | | | | | | | |
| pt10 | pre | 12 | 1 | 0 | 89 | 19 | 12 |
| | max | 109 | 21 | 0 | 1344 | 93 | 52 |
| pt11 | pre | 4 | 5 | 0 | 39 | 68 | 2 |
| | max | 44 | 18 | 0 | 1578 | 391 | 74 |
| pt12 | pre | 0 | 0 | 3 | nt | nt | nt |
| | max | 53 | 61 | 0 | 860 | 229 | 4 |
| pt13 | pre | 7 | 8 | 0 | 90 | 44 | 10 |
| | max | 8 | 27 | 1 | 314 | 279 | 143 |

Max: maximal effect on immunological parameter during rIL-2 treatment. In all but one patient (pt 9) this effect was maximal at the end of the treatment. The cytotoxic capacity of the patients' PBL is expressed in Lytic Units (30%)/$10^6$ effector cells, where one Lytic Unit is defined as the number of effector cells that produce 30% lysis of 1000 target cells. nt = not tested.

**Table IV** Proliferative capacity of the isolated PBL from patients in groups I–IV

| *Patient* | | *Medium* | *6 rIL-2 21 pM* | *600 rIL-2 2.1 nM* | *αCD28* | *αCD3* | *αCD3+ αCD28* |
|---|---|---|---|---|---|---|---|
| *Group I* | | | | | | | |
| pt 1 | pre | <1 | 9 | 11 | 1 | 18 | 96 |
| | max | <1 | 5 | 10 | 2 | 22 | 91 |
| pt 2 | pre | <1 | 14 | 22 | 11 | 17 | 80 |
| | max | <1 | 12 | 14 | 5 | 15 | 76 |
| *Group II* | | | | | | | |
| pt 6 | pre | <1 | 4 | 7 | <1 | 28 | 84 |
| | max | <1 | 7 | 8 | <1 | 50 | 95 |
| *Group III* | | | | | | | |
| pt 7 | pre | <1 | 8 | 21 | 2 | 70 | 83 |
| | max | <1 | 7 | 23 | 1 | 57 | 94 |
| pt 8 | pre | <1 | 2 | 3 | 25 | 3 | 44 |
| | max | <1 | 2 | 2 | 23 | 2 | 50 |
| pt 9 | pre | <1 | 2 | 4 | <1 | 15 | 51 |
| | max | <1 | 4 | 6 | 3 | 26 | 89 |
| *Group IV* | | | | | | | |
| pt 10 | pre | <1 | 5 | 12 | 4 | 31 | 132 |
| | max | <1 | 4 | 36 | <1 | 14 | 129 |
| pt 11 | pre | <1 | 4 | 10 | 4 | 16 | 85 |
| | max | <1 | 12 | 27 | <1 | 5 | 80 |
| pt 13 | pre | <1 | 5 | 6 | <1 | nt | nt |
| | max | <1 | 9 | 7 | 1 | nt | nt |

Max: maximal effect on immunological parameter during rIL-2 treatment. In all but one patient (pt 9) this effect was maximal at the end of treatment. αCD28 = anti-CD28 (1/1000) antibody; αCD3 = immobilised CD3 (1/2000) antibody. Data are expressed as mean c.p.m. × $10^{-3}$ from triplicate cultures. nt = not tested.

*$6 \times 10^6$ IU $m^{-2}$ 24 $h^{-1}$ (group IV)*

Three patients were treated at this dose level for 7, 14 and 18 weeks respectively. Pt 13 was initially treated with $3 \times 10^6$ IU $ml^{-2}$ 24 $h^{-1}$ and after 7 weeks the dose was escalated to $6 \times 10^6$ IU $m^{-2}$ 24 $h^{-1}$ for another 7 weeks. During the first weeks of treatment no consistent changes in the lymphocyte count were observed but after prolonged treatment a sustained increase of the lymphocyte count was noted. In patient 10 the dose was escalated to $9 \times 10^6$ IU $m^{-2}$ 24 $h^{-1}$ after 10 weeks of treatment without evident effect on the lymphocyte count. In pt 13 no changes in the total lymphocyte count were observed, not even after dose escalation.

No changes in the monocyte count occurred in any of the patients. Since the most striking immunological effects were noted in these patients, these results will be discussed in detail.

*A: Immunophenotyping of lymphocyte subpopulations (group IV)*

In all three patients treated at the dose level of $6 \times 10^6$ IU $m^{-2}$ 24 $h^{-1}$ there was a pronounced shift within the lymphocyte population. The percentage of $CD3^+$ (T) cells decreased to about one quarter of the baseline levels, while the absolute number of $CD3^+$ cells remained the same throughout the treatment (Table II). The percentage and absolute number of $CD56^+$ cells showed a dramatic and sustained increase during treatment. Figure 1 shows a representative graph of the changes in numbers of $CD3^+$ and $CD56^+$ lymphocytes. After treatment the number of $CD3^+$ and $CD56^+$ cells returned to pre-treatment values. Within the $CD56^+$ cell compartment, a preferential increase in the percentage of cells with high expression of CD56 ($CD56^{bright}$) was noted (Figure 2). In addition there was a preferential increase in the percentage of cells with low expression of CD16 ($CD16^{dim}$) and of CD8 ($CD8^{dim}$) cells (Figure 2).

**Figure 1** Total lymphocyte count and the numbers of ($CD3^+$) T lymphocytes and ($CD56^+$) natural killer cells in patient 11 treated with $6 \times 10^6$ IU $m^{-2}$ 24 $h^{-1}$ rIL-2.

The ratio of $CD4^+$ and $CD8^{bright}$ lymphocytes did not change in any of the patients during treatment, while the relative and absolute number of cells expressing CD25 ($\alpha$ chain of the IL-2 receptor) increased to a variable extent. The number of cells expressing p75 ($\beta$ chain of the IL-2 receptor) substantially increased in all patients during treatment and correlated closely with the number of $CD56^+$ cells ($r = 0.966$). Simultaneously, the mean fluorescence of CD38 increased (data not shown).

A close correlation was found between the number of $CD3^+$ and the number of $CD28^+$, and $CD45RO^+$ cells (data not shown). No changes in cellular expression of CD27, CD28 and CD45RO were observed.

After discontinuation of treatment, all parameters returned to pre-treatment level.

No significant changes in the number of B cells were noted. The expression of the adhesion molecules CD11a, CD18, CD58 (LFA-3) and CD54 (ICAM-1) was studied on the lymphocytes of two patients (pts 10 and 11). CD11a/CD18 and CD58 were present on essentially all lymphocytes. There was a tendency to increased expression of CD11a/CD18 during treatment, whereas no changes were seen in the expression of CD58. The activation epitope of LFA-1$\alpha$ chain, detected by the monoclonal antibody NKI-L16, showed no consistent changes in the total lymphocyte population. The number of $CD54^+$ lymphocytes, determined on a limited number of samples, increased during treatment (data not shown).

In patient 10 the escalation of the dose from 6 to $9 \times 10^6$ IU $m^{-2}$ 24 $h^{-1}$ in week 10 had no evident effect on the lymphocyte populations. Within the lymphocyte population of patient 13 a relative increase of NK cells was only observed after the dose escalation to $6 \times 10^6$ IU $m^{-2}$ $h^{-1}$.

*Two colour fluorescence (group IV)*

A number of phenotypic markers was also tested by double staining, in order to discriminate between their expression on T cells and on NK cells. In patient 10 the week 6, 10 and 12 samples were analysed. In patient 11 double staining was performed on samples taken before (week 0) and at week 6, 12, 15 and 18 during treatment while in patient 13 samples taken before (week 0) and at week 9 were available.

**Figure 2** Changes in expression of CD56, CD16 and CD8 on the PBL from patient 10 (above) and patient 11 (below) before treatment (thin lines) and during treatment at 14 weeks (pt 10) and 12 weeks (pt 11) respectively (thick lines). The figures show a definite increase in the relative expression of $CD56^{bright}$, $CD16^{dim}$, and $CD8^{dim}$ cells in both patients.

Only a small percentage (5%) of the PBL were CD3$^+$ CD56$^+$ throughout rIL-2 treatment.

Within the NK cell population significant changes occurred after $\geqslant$6 weeks of treatment, consisting of an increase of CD56$^{bright}$ cells in all patients. After $\geqslant$6 weeks of treatment 30–85% of the entire CD56$^+$ cells expressed CD16, 35–80% expressed CD8, $\geqslant$95% expressed p75 and CD2, virtually no cells expressed CD25, and 30–45% expressed CD27. The adhesion molecule LFA-1 was present on virtually all CD56$^+$ cells, and 60–75% expressed CD54 (ICAM-1). There was a substantial difference in the level of expression of some of these antigens among the CD56$^{dim}$ and CD56$^{bright}$ subpopulations. In comparison with CD56$^{dim}$ cells, CD56$^{bright}$ cells had a low expression of CD16, while the expression of CD2, CD27 and CD54 was higher on these cells. Between the two NK subsets there was no difference in the level of p75 expression.

Only pt 11 had a sufficient number of CD56$^+$ cells before treatment to perform a reliable double staining. The expression of the various antigens on CD56$^+$ cells before and at weeks 15 of treatment were tested on two occasions. During treatment the percentage of CD56$^+$ cells expressing CD8 increased from 60 to 70%, those expressing p75 rose from 80 to 95%, those expressing CD54 from 35 to 55% and those expressing CD27 from 20 to 45%, while the percentage of CD56$^+$ expressing CD16 (80–85%), CD2 (80–95%) and CD25 (<2%) remained similar (mean values of the two experiments). Representative results are depicted in Figure 3a illustrating that the CD56$^{bright}$ cells express CD16 at a low level, and CD54, CD2 and CD27 at a high level. To investigate the hypothesis that CD27 is primarily expressed on CD56$^{bright}$CD16$^{dim}$ NK cells, we examined the expression of CD27 on CD16$^+$ cells, and demonstrated that CD27 is indeed primarily expressed on CD16$^{dim}$ cells (Figure 3b).

No changes occurred within the T cell population. Before and during treatment 15–40% of the CD3$^+$ population were CD8$^{bright}$, 15–45% expressed CD25. Nearly all T cells expressed CD27 and CD2, while p75 was virtually negative. LFA-1 was present on virtually all CD3$^+$ cells, while <30% of the CD3$^+$ lymphocytes expressed CD54 (tested on a limited number of samples in pts 10 and 11).

*B: Cytotoxic capacity (group IV)*

*Cytotoxicity of PBL without* in vitro *rIL-2 stimulation* In patients 10, 11 and 12 the PBL showed a considerable increase in NK and ADCC activity during treatment (Table III). The time-course of this increase in cytotoxic capacity is shown in Figure 4. Cytotoxicity only became apparent in the samples taken after 6 weeks of treatment and then remained elevated throughout the entire treatment period.

To exclude the possibility that the increased cytotoxic capacity was solely the result of an increased number of NK cells, we also expressed the results in Lytic Units $10^{-6}$ CD56$^+$ cells. After this re-calculation the cytotoxic capacity of the PBL sampled during treatment remained enhanced. These results suggest that enhanced activation of NK cells is induced by the rIL-2 treatment. The limited number of PBL precluded cytotoxicity assays after T cell depletion.

In patient 13, ADCC was only observed after dose escalation.

**Figure 3** **a,** Double staining of PBL from patient 11 before (left) and after 15 weeks of rIL-2 treatment (right). Figure 3a shows that after treatment there is an increase in the percentage of CD56$^{bright}$ cells. The percentage of CD56$^+$ cells that express p75, CD54 and CD27 is markedly increased, while the percentage of CD56$^+$ cells expressing CD16 and CD2 is not evidently altered. The graphs also indicate that C56$^{bright}$ cells have a low level of CD16, and a high expression of CD54, CD2 and CD27, but not of p75. **b,** Demonstrates that CD27 is primarily present on CD16$^{dim}$ cells.

**Figure 4** The cytotoxic capacity of the PBL from three patients (pts 10, 11 and 12) treated at the highest dose level. The PBL were cultured overnight in medium without rIL-2 and the capacity of the PBL to lyse K562 was determined in a standard $^{51}Cr$ release assay (NK activity). Solid bars represent the cytotoxic capacity of the isolated peripheral blood lymphocytes (PBL), hatched bars represent the cytotoxic capacity of the isolated PBL after correction for the number of $CD56^+$ natural killer cells (CD56).

*Cytotoxic capacity of the PBL after* in vitro *stimulation with rIL-2* In the two patients (10 and 11) tested, $NK_a$, $LAK_a$ and $ADCC_a$ were enhanced during treatment, while in patient 13 these cytotoxic activities increased only after dose escalation (Table III).

*C:* In vitro *proliferation assay (Group IV)*

The PBL of two (pts 10 and 11) of the three tested patients showed an increased response to 600 IU rIL-2 $ml^{-1}$, while one of them (pt 11) even responded to 6 IU rIL-2 $ml^{-1}$ (Table IV), suggesting the presence of lymphocytes expressing the high-affinity IL-2 receptor.

No enhanced proliferation was noted upon *in vitro* stimulation with CD3 and/or CD28 antibodies in any of the patients. The reduced response to anti-CD3 in patients 10 and 11 may be related to a diminished percentage of T cells during treatment.

*Serum anti IL-2 antibodies and soluble IL-2 receptor (groups I–IV)*

Before and after treatment no IgG and IgM antibodies against IL-2 were detected.

The plasma level of the soluble IL-2 receptor (sIL-2R) was determined on cryopreserved plasma isolated before, at regular intervals during and after rIL-2 treatment. Before and after treatment the plasma sIL-2R concentration was below 200 U $ml^{-1}$. There was a close relationship ($r = 0.948$) between the dose and the plasma sIL-2R concentration as measured after 3 and 6 weeks of rIL-2 treatment. During further prolonged rIL-2 treatment the plasma sIL-2R tended to decrease. After dose escalation, no increase in the plasma sIL-2 was noted. There was no correlation between the plasma sIL-2R concentration and the absolute number of $CD25^+$ cells ($r = 0.204$) or any other immunological parameter.

### Discussion

In this study we investigated the immuno-modulating effects of prolonged continuous infusion of EuroCetus rIL-2 and demonstrated that this mode of administration for a period of more than 6 weeks may result in a sustained activation of the immunesystem. We confirm the data from others that prolonged treatment with rIL-2 results primarily in an increased number and activity of NK cells in a dose-dependent way (Caligiuri *et al.*, 1991; Soiffer *et al.*, 1992).

The most striking and consistent immunological effects occurred at a dose $6 \times 10^6$ IU $m^{-2}$ 24 $h^{-1}$. In these patients we noted a sustained and marked increase of NK cells with enhanced cytotoxic capacity of the PBL, without alterations within the T cell subpopulations.

Particularly in the two patients treated for 14 and 18 weeks, a steady time-dependent lymphocytosis occurred with a 10–20 fold increase in the number of natural killer cells. Natural killer cells, phenotypically best defined as $CD3^-$ $CD56^+$ lymphocytes, comprise 10–15% of normal resting blood lymphocytes (Nagler *et al.*, 1989; Lanier *et al.*, 1986; Robertson *et al.*, 1990*b*). Based on the cellular co-expression of CD56 and CD16 (the low affinity Fc receptor for IgG), several subsets of NK cells can be identified. More than 90% of resting NK cells express CD56 at a low level with a high expression of CD16 ($CD56^{dim}CD16^{bright}$), and a small subset of NK cells express CD56 at a high level ($CD56^{bright}$) with low or no expression of CD16 (Nagler *et al.*, 1989; Lanier *et al.*, 1986). This and other studies indicate that rIL-2 treatment leads to a preferential increase in the relative and absolute number of the $CD56^{bright}CD16^{dim}$ and $CD56^{bright}$-$CD16^-$ subsets (Ellis *et al.*, 1988; 1989; Soiffer *et al.*, 1992; Weil-Hillman *et al.*, 1989; Urba *et al.*, 1990).

The shift within the NK subsets has several consequences for the phenotypic and functional characteristics of the NK cells. The intermediate affinity IL-2 receptor p75 is expressed by the majority of resting NK cells (Caligiuri *et al.*, 1990; Nagler *et al.*, 1989; Ohashi *et al.*, 1989; Tsudo *et al.*, 1986; Voss *et al.*, 1990) and only a small subpopulation of NK (the $CD56^{bright}$) cells express the high-affinity IL-2 receptor (CD25/p75) (Caligiuri *et al.*, 1990; Nagler *et al.*, 1990). We and others (Thompson *et al.*, 1989; Voss *et al.*, 1990; Weil-Hillman *et al.*, 1990) have demonstrated that the CD25 expression on $CD56^+$ cells remains virtually negative during prolonged *in vivo* rIL-2 treatment, suggesting down regulation of the high-affinity IL-2 receptor on the $CD56^{bright}$ NK subset (Voss *et al.*, 1990). Nevertheless, in patient 11 the appearance of a small subset of NK cells expressing the high affinity IL-2 receptor is suggested by the fact that the PBL of this patient displayed an enhanced proliferative response upon *in vitro* stimulation with 6 IU $ml^{-1}$ rIL-2 (Weil-Hillman *et al.*, 1989; 1990).

During rIL-2 treatment we and others observed an increase of $CD56^+$ cells expressing the adhesion molecule CD54 (ICAM-1) (Triozzi *et al.*, 1992), indicating activation as has been demonstrated after *in vitro* stimulation (Robertson *et al.*, 1990*a*). We noted that the increase occurred primarily on the $CD56^{bright}CD16^{dim}$ cells.

Unexpectedly, we found that prolonged rIL-2 treatment resulted in an increased expression of CD27 on $CD56^+$ cells, particularly on the $CD56^{bright}CD16^{dim}$ population. CD27, initially thought to be a T cell-specific antigen present on the majority of T cells, plays a prominent role in T cell activation (Lier van *et al.*, 1987; Sugita *et al.*, 1991). Very recently it has been demonstrated that CD27 is also expressed at a low level on resting NK cells, especially on the $CD56^{bright}$ population and that the expression is upregulated after *in vitro* stimulation with rIL-2 (Sugita *et al.*, 1992). Our data confirm that CD27 is present on a small percentage of resting NK cells and show that CD27 is upregulated on a subpopulation after *in vivo* rIL-2 treatment.

These phenotypic changes, reflecting the presence of an activated NK cell population induced by rIL-2 treatment, were accompanied by an increased natural killer (NK) activity and antibody dependent cellular cytotoxicity (ADCC), which was further enhanced after additional *in vitro* stimulation with rIL-2. Lymphokine activated killer (LAK) activity measured after *in vitro* rIL-2 stimulation was also increased during treatment. Increased cytotoxic capacity of PBL during rIL-2 treatment has been described by others using freshly isolated cells (Creekmore *et al.*, 1989; Kohler *et al.*, 1989; Thompson *et al.*, 1989; Urba *et al.*, 1990). Because we used cryopreserved lymphocytes in order to test all samples in a single experiment, no quantitative comparison with our data can be made.

Since T cell activation, determined by an increase of $CD3^+$ cells expressing CD25 or HLA-DR, has only been observed after short-term treatment with high-dose rIL-2 (Thompson *et al.*, 1989; Yoshino *et al.*, 1991), it is not surprising that we did not find indications for T cell expansion or activation after prolonged infusion of relatively low dose rIL-2. The $CD4/CD8^{bright}$ ratio remained unchanged during treatment and there was no increased expression of markers associated with T cell activation, such as CD27 or CD25. One patient (pt 9), however, treated at $1.8 \times 10^6$ IU $m^{-2}$ 24 $h^{-1}$ had subtle signs of T cell activation after 3 weeks of treatment. A low expression of the high-affinity IL-2 (CD25/p75) was present on $CD3^+$ cells, the isolated PBL showed increased proliferation upon T cell specific stimulation, and the patient had disproportionally elevated serum soluble IL-2 receptor levels, which may be indicative for lymphocyte activation (Voss *et al.*, 1989; Rubin *et al.*, 1990).

The observed pattern of the serum soluble IL-2 receptor levels in our patients is in agreement with that observed by others (Bogner *et al.*, 1992; Lissoni *et al.*, 1991: Lotze *et al.*, 1987; Voss *et al.*, 1989) showing that during prolonged intermittent or continuous rIL-2 treatment, the serum levels gradually increase during the first week(s) followed by a plateau level or a gradual decline as rIL-2 treatment continues. Although *in vitro* studies indicate that soluble IL-2 receptor may be released upon stimulation of T and B lymphocytes (Hofmann *et al.*, 1992), the source of sIL-2R in patients treated with rIL-2 for advanced cancer is obscure. In this study we confirm that the serum sIL-2R levels are closely related to the dose of rIL-2 (Bogner *et al.*, 1992). As also reported by others (Lotze *et al.*, 1987), the serum sIL-2R level could be correlated with the clinical toxicity observed in our patients (Vlasveld *et al.*, 1992), which reached a peak after 3 weeks of treatment followed by a decrease despite continuation of the rIL-2 treatment.

In the clinical analysis of the present study (Vlasveld *et al.*, 1992) a short term partial remission was observed in one patient (pt 8). The immunological effects in this patient were not different from those observed in non responding patients.

In conclusion, prolonged continuous intravenous administration of rIL-2 results in a dose-dependent increase in the number of NK cells, preferentially of the $CD56^{bright}CD16^{dim}$ fraction. The NK cells had phenotypical signs of activation and displayed enhanced functional activity. During treatment we observed no expansion or activation of T cells. In contrast to the observed transient pattern of clinical toxicity and eosinophilia (Vlasveld *et al.*, 1992), the changes within the NK population were sustained.

### References

BOGNER, M.P., VOSS, S.D., BECHHOFER, R., HANK, J.A., ROPER, M., POPLACK, D., HAMMOND, D. & SONDEL, P.M. (1992). Serum CD25 levels during interleukin-2 therapy: dose dependence and correlations with clinical toxicity and lymphocyte surface sCD25 expression. *J. Immunother.*, **11**, 111–118.

CALIGIURI, M.A., MURRAY, C., SOIFFER, R.J., KLUMPP, T.R., SEIDEN, M., COCHRAN, K., CAMERON, C., ISH, C., BUCHANAN, L., PERILLO, D., SMITH, K. & RITZ, J. (1991). Extended continuous infusion low-dose recombinant interleukin-2 in advanced cancer: prolonged immunomodulation without significant toxicity. *J. Clin. Oncol.*, **9**, 2110–2119.

CALIGIURI, M.A., ZMUIDZINAS, A., MANLEY, T.J., LEVINE, H., SMITH, K.A. & RITZ, J. (1990). Functional consequences of interleukin 2 receptor expression on resting human lymphocytes. *J. Exp. Med.*, **171**, 1509–1526.

CREEKMORE, S.P., HARRIS, J.E., ELLIS, T.M., BRAUN, D.P., COHEN, I.I., BHOOPALAM, N., JASSAK, P.F., CAHILL, M.A., CANZONERI, C.L. & FISHER, R.I. (1989). A phase I clinical trial of recombinant interleukin-2 by periodic 24 hour intravenous infusions. *J. Clin. Oncol.*, **7**, 276–284.

ELLIS, T.M., CREEKMORE, S.P., MCMANNIS, J.D., BRAUN, D.P., HARRIS, J.A. & FISHER, R.I. (1988). Appearance and phenotypic characterization of circulating leu19+ cells in cancer patients receiving recombinant interleukin 2. *Cancer Res.*, **48**, 6597–6602.

ELLIS, T.M. & FISHER, R.I. (1989). Functional heterogeneity of leu $19^{'bright'+}$ and leu $19^{'dim'+}$ lymphokine-activated killer cells. *J. Immunol.*, **142**, 2949–2954.

GAMBACORTI-PASSERINI, C., RADRIZZANI, M., MAROLDA, R., BELLI, F., SCIORELLI, G., GALAZKA, A.R., SCHINDLER, J.D., CASCINELLI, N. & PARMIANI, G. (1988). *In vivo* activation of lymphocytes in melanoma patients receiving escalating doses of recombinant interleukin 2. *Int. J. Cancer*, **41**, 700–706.

GEMLO, B.T., PALLADINO, M.A., JAFFE, H.S., ESPEVIK, T.P. & RAYNER, A.A. (1988). Circulating cytokines in patients with metastatic cancer treated with recombinant interleukin 2 and lymphokine-activated killer cells. *Cancer Res.*, **48**, 5864–5867.

GRIMM, E.A., MAZUMBER, E.A., ZHANG, H.Z. & ROSENBERG, S.A. (1982). Lymphokine activated killer cell phenomenon: lysis of natural killer-resistant fresh solid tumor cells by interleukin-2 activated autologous human peripheral blood lymphocytes. *J. Exp. Med.*, **155**, 1823–1841.

HANK, J.A., KOHLER, P.C., WEIL-HILLMAN, G., ROSENTHAL, N., MOORE, K.H., STORER, B., MINKOFF, D., BRADSHAW, J., BECHHOFER, R. & SONDEL, P.M. (1988). *In vivo* induction of the lymphokine-activated killer phenomenon: interleukin 2-dependent human non-major histocompatibility complex-restricted cytotoxicity generated *in vivo* during administration of human recombinant interleukin 2. *Cancer Res.*, **48**, 1965–1971.

HANK, J.A., ROBINSON, R.R., SURFUS, J., MUELLER, B.M., REISFELD, R.A., CHEUNG, N.-K. & SONDEL, P.M. (1990). Augmentation of antibody dependent cell mediated cytotoxicity following *in vivo* therapy with recombinant interleukin 2. *Cancer Res.*, **50**, 5234–5239.

HENNY, C.S., KURIBAYASHI, K., KERN, D.E. & GILLIS, S. (1981). Interleukin-2 augments natural killer cell activity. *Nature*, **291**, 335–338.

HOFMANN, B., BASS, H., NISHANIAN, P., FAISAL, M., FIGLIN, R.A., SARNA, G.P. & FAHEY, J.L. (1992). Different lymphoid cell populations produce varied levels of neopterin, β2-microglobulin and soluble IL-2 receptor when stimulated with IL-2, interferon-gamma or tumour necrosis factor-alpha. *Clin. Exp. Immunol.*, **88**, 548–554.

KOHLER, P.C., HANK, J.A., MOORE, K.H., STORER, B., BECHHOFER, R., HONG, R. & SONDEL, P.M. (1989). Phase 1 clinical trial of recombinant interleukin-2: a comparison of bolus and continuous infusion. *Cancer Invest.*, **7**, 213–223.

LANIER, L.L., MY LE, A., CIVIN, C.I., LOKEN, M.R. & PHILIPS, J.H. (1986). The relationship of CD16 (leu 11) and leu 19 (NKH-1) antigen expression on human peripheral blood NK cells and cytotoxic T lymphocytes. *J. Immunol.*, **136**, 4480–4486.

LIANG, S.-M., ALLET, B., ROSE, K., HIRSCHI, M., LIANG, C.-M. & THATCHER, D.R. (1985). Characterization of human interleukin 2 derived from *Escherichia coli*. *Biochem. J.*, **229**, 429–439.

LIER VAN, R.A.W., BORST, J., VROOM, T.M., KLEIN, H., MOURIK VAN, P., ZEYLEMAKER, W.P. & MELIEF, C.J.M. (1987). Tissue distribution and biochemical and functional properties of Tp55 (CD27). A novel T cell differentiation antigen. *J. Immunol.*, **139**, 1589–1596.

LISSONI, P., TISI, E., BRIVIO, F., BARNI, S., ROVELLI, F., PEREGO, M. & TANCINI, G. (1991). Increase in soluble interleukin-2 receptor and neopterin serum levels druing immunotherapy of cancer with interleukin-2. *Eur. J. Cancer*, **27**, 1014–1016.

LOTZE, M.T., CUSTER, M.C., SHARROW, S.O., RUBIN, L.A., NELSON, D.L. & ROSENBERG, S.A. (1987). *In vivo* administration of purified human interleukin-2 to patients with cancer: development of interleukin-2 receptor positive cells and circulating soluble interleukin-2 receptor following interleukin-2 administration. *Cancer Res.*, **47**, 2188–2195.

LOTZE, M.T., GRIMM, E.A., MAZUMBER, A., STRAUSSER, J.L. & ROSENBERG, S.A. (1981). Lysis of fresh and cultured autologous tumor by human lymphocytes cultured in T-cell growth factor. *Cancer Res.*, **41**, 4420–4425.

LOTZE, M.T., MATORY, Y.L., ETTINGHAUSEN, S.E., RAYNER, A.A., SHARROW, S.O., SEIPP, C.A.Y., CUSTER, M.C. & ROSENBERG, S.A. (1985). *In vivo* administration of purified human interleukin 2: II: Half-life, immunologic effects, and expansion of peripheral lymphoid cells *in vivo* with recombinant IL-2. *J. Immunol.*, **135**, 2865–2875.

MALKOVSKY, M., LOVELAND, B., NORTH, M., ASHERSON, G.L., GAO, L., WARD, P. & FIERS, W. (1987). Recombinant interleukin-2 directly augments the cytotoxicity of human monocytes. *Nature*, **325**, 262–265.

MORGAN, D., RUSCETTI, F.W. & GALLOW, R. (1976). Selective *in vitro* growth of T-lymphocytes from normal human bone marrows. *Science*, **193**, 1007–1008.

NAGLER, A., LANIER, L.L. & PHILIPS, J.H. (1990). Constitutive expression of high affinity interleukin 2 receptors on human CD16$^-$ natural killer cells *in vivo*. *J. Exp. Med.*, **171**, 1527–1533.

NAGLER, A., LANIER, L.L., CWIRLA, S. & PHILIPS, J.H. (1989). Comparative studies of human FcRIII-positive and negative natural killer cells. *J. Immunol.*, **143**, 3183–3191.

NEGRIER, S., PHILIP, T., STOTER, G., FOSSA, S.D., JANSSEN, S., IACONE, A., CLETON, F.S., EREMIN, O., ISREAL, L., JASMIN, C., RUGARLI, C., MASSE, H.V.D., THATCHER, N., SYMANN, M., BARTSCH, H.H., BERGMANN, L., BIJMAN, J.T., PALMER, P.A. & FRANKS, C.R. (1989). Interleukin-2 with or without LAK cells in metastatic renal cell carcinoma: a report of a European multicentre study. *Eur. J. Cancer Clin. Oncol.*, **25** (Suppl. 3), 21–28.

NIHUIS, E.W.P., WIEL VAN DE-KEMENADE VAN, E., FIGDOR, C.G. & LIER VAN, R.A.W. (1990). Activation and expansion of tumour-infiltrating lymphocytes by anti-CD3 and anti-CD28 monoclonal antibodies. *Cancer Immunol. Immunother.*, **32**, 245–250.

OHASHI, Y., TAKASHITA, T., NAGATA, K., MORI, S. & SUGAMURA, K. (1989). Differential expression of the IL-2 receptor subunits, p55 and p75 on various populations of primary peripheral blood mononuclear cells. *J. Immunol.*, **143**, 3548–3555.

PROSS, H.F., BAINES, M.G., RUBIN, P., SCHRAGGE, P. & PATTERSON, M.S. (1981). Spontaneous human lymphocyte-mediated cytotoxicity against tumor target cells. IX. The quantitation of natural killer cell activity. *J. Clin. Immunol.*, **1**, 51–63.

ROBERTSON, M.J., CALIGIURI, M.A., MANLEY, T.J., LEVINE, H. & RITZ, J. (1990*a*). Human natural killer cell adhesion molecules: differential expression after activation and participation in cytolysis. *J. Immunol.*, **145**, 3194–3201.

ROBERTSON, M.J. & RITZ, J. (1990*b*). Biology and clinical relevance of human natural killer cells. *Blood*, **76**, 2421–2438.

ROSENBERG, S.A., GRIMM, E.A., MCGROGAN, M., DOYLE, M., KAWASAKI, E., KOTHS, K. & MARK, D.F. (1984). Biological activity of recombinant human interleukin-2 produced in *Eschericia coli*. *Science*, **223**, 1412–1415.

ROSENBERG, S.A., LOTZE, M.T., YANG, J.C., AEBERSOLD, P.M., LINEHAN, W.M., SEIPP, C.A. & WHITE, D.E. (1989). Experience with the use of high-dose interleukin-2 in the treatment of 652 cancer patients. *Ann. Surg.*, **210**, 474–485.

RUBIN, L.A. & NELSON, D.L. (1990). The soluble interleukin-2 receptor: biology, function, and clinical application. *Ann. Inter. Med.*, **113**, 619–627.

SCHAAFSMA, M.R., FALKENBURG, J.H.F., LANDEGENT, J.E., DUINKERKEN, N., OSANTO, S., RALPH, P., KAUSHANSKY, K., WAGEMAKER, G., DAMME VAN, J., WILLEMZE, R. & FIBBE, W.E. (1991). *In vivo* production of interleukin-5, granulocyte-macrophage colony-stimulating factor, macrophage colony-stimulating factor, and interleukin-6 during intravenous administration of high-dose interleukin-2 in cancer patients. *Blood*, **78**, 1981–1987.

SOIFFER, R.J., MURRAY, C., COCHRAN, K., CAMERON, C., WANG, E., SCHOW, P.W., DALEY, J.F. & RITZ, J. (1992). Clinical and immunologic effects of prolonged infusion of low-dose recombinant interleukin-2 after autologous and T-cell-depleted allogeneic bone marrow transplantation. *Blood*, **79**, 517–526.

SONDEL, P.M., KOHLER, P.C., HANK, J.A., MOORE, K.H., ROSENTHAL, N.S., SOSMAN, J.A., BECHHOFER, R. & STORER, B. (1988). Clinical and immunological effects of recombinant interleukin 2 given by repetitive weekly cycles to patients with cancer. *Cancer Res.*, **48**, 2561–2567.

SUGITA, K., TORIMOTO, Y., NOJIMA, Y., DALEY, J.F., SCHLOSSMAN, S.F. & MORIMOTO, C. (1991). The 1A4 molecule (CD27) is involved in T cell activation. *J. Immunol.*, **147**, 1477–1483.

SUGITA, K., ROBERTSON, M.J., TORIMOTO, Y., RITZ, J., SCHLOSSMAN, S.F. & MORIMOTO, C. (1992). Participation of the CD27 antigen in the regulation of IL-2-activated human natural killer cells. *J. Immunol.*, **149**, 1199–1203.

TAKESHITA, T., GOTO, Y., TADA, K., NAGATA, K., ASAO, H. & SUGAMURA, K. (1989). Monoclonal antibody defining a molecule possibly identical to the p75 subunit of interleukin 2 receptor. *J. Exp. Med.*, **169**, 1323–1332.

TANIGUCHI, T., MATSUI, H., FUIJITA, T., TAKAOKA, C., KASHIMA, N., YOSHIMOTO, R. & HAMURO, J. (1983). Structure and expression of a cloned cDNA for human interleukin-2. *Nature*, **302**, 305–310.

THOMPSON, J.A., LEE, D.J., LINDGREN, C.G., BENZ, L.A., COLLINS, C., LEVITT, D. & FEFER, A. (1988). Influence of dose and duration of infusion of interleukin-2 on toxicity and immunomodulation. *J. Clin. Oncol.*, **6**, 669–678.

THOMPSON, J.A., LEE, D.J., LINDGREN, C.G., BENZ, L.A., COLLINS, C., SHUMAN, W.P., LEVITT, D. & FEFER, A. (1989). Influence of schedule of interleukin 2 administration on therapy with interleukin 2 and lymphokine activated killer cells. *Cancer Res.*, **49**, 235–240.

TRIOZZI, P.L., EICHER, D.M. & RINEHART, J.J. (1992). Modulation of adhesion molecules on human large granular lymphocytes by interleukin-2 *in vivo* and *in vitro*. *Cell Immunol.*, **140**, 295–303.

TSUDO, M., KITAMURA, F. & MIYASAKA, M. (1989). Characterization of the interleukin 2 receptor $\beta$ chain using three distinct monoclonal antibodies. *Proc. Natl Acad. Sci. USA*, **86**, 1982–1986.

URBA, W.J., STEIS, R.G., LONGO, D.L., KOPP, W.C., MALUISH, A.E., MARCON, L., NELSON, D.L., STEVENSON, H.C. & CLARK, J.W. (1990). Immunomodulatory properties and toxicity of interleukin 2 in patients with cancer. *Cancer Res.*, **50**, 185–192.

VLASVELD, L.T., RANKIN, E.M., HEKMAN, A., RODENHUIS, S., BEIJNEN, J.H., HILTON, A.M., DUBBELMAN, A.C., VYTH-DREESE, F.A. & MELIEF, C.J.M. (1992). A phase I study of prolonged continuous infusion of low dose recombinant interleukin-2 in melanoma and renal cell cancer. Part I: clinical aspects. *Br. J. Cancer*, **65**, 744–500.

VOSS, S.D., HANK, J.A., NOBIS, C.A., FISCH, P., SOSMAN, J.A. & SONDEL, P.M. (1989). Serum levels of low-affinity interleukin-2 receptor molecule (TAC) during IL-2 therapy reflect systemic lymphoid activation. *Cancer Immunol. Immunother.*, **29**, 261–269.

VOSS, S.D., ROBB, R.J., WEIL-HILLMAN, G., HANK, J.A., SUGAMURA, K., TSUDO, M. & SONDEL, P.M. (1990). Increased expression of the interleukin 2 (IL-2) receptor $\beta$ chain (p70) on CD56$^+$ natural killer cells after *in vivo* IL-2 therapy: p70 expression does not alone predict the level of intermediate affinity IL-2 binding. *J. Exp. Med.*, **172**, 1101–1114.

VYTH-DREESE, F.A., REIJDEN VAN DER, H.J. & VRIES DE, J.E. (1982). Phorbol-ester-mediated induction and augmentation of mitogenesis and interleukin-2 production in human T-cell lymphoproliferative disease. *Blood*, **60**, 1437–1446.

WALDMANN, T.A., GOLDMAN, C.K., ROBB, R.J., DEPPER, J.M., LEONARD, W.J., SHARROW, S.O., BONGIOVANNI, K.F., KORSMEYER, S.J. & GREENE, W.C. (1984). Expression of interleukin-2 receptors on activated human B cells. *J. Exp. Med.*, **160**, 1450–1466.

WEIL-HILLMAN, G., FISCH, P., PRIEVE, A.F., SOSMAN, J.A., HANK, J.A. & SONDEL, P.M. (1989). Lymphokine-activated killer activity induced by *in vivo* interleukin 2 therapy: predominant role for lymphocytes with increased expression of CD2 and leu 19 antigens but negative expression of CD16 antigens. *Cancer Res.*, **49**, 3680–3688.

WEIL-HILLMAN, G., VOSS, S.D., FISCH, P., SCHELL, K., HANK, J.A., SOSMAN, J.A., SUGAMURA, K. & SONDEL, P.M. (1990). Natural killer cells activated by interleukin 2 treatment *in vivo* respond to interleukin 2 primarily through the p75 receptor and maintain the p55 (TAC) negative phenotype. *Cancer Res.*, **50**, 2683–2691.

YOSHINO, I., YANO, T., MURATA, M., ISHIDA, T., SUGIMACHI, K., KIMURA, G. & NOMOTO, K. (1991). Cytolytic potential of peripheral blood T-lymphocytes following adoptive immunotherapy with lymphokine-activated killer cells and low-dose interleukin 2. *Cancer Res.*, **51**, 1494–1498.